# The Genetic Epidemiology of Joint Shape and the Development of Osteoarthritis

**DOI:** 10.1007/s00223-020-00702-6

**Published:** 2020-05-11

**Authors:** J. Mark Wilkinson, Eleftheria Zeggini

**Affiliations:** 1 Department of Oncology and Metabolism, University of Sheffield, Sheffield, UK; 2 Institute of Translational Genomics, Helmholtz Zentrum München – German Research Center for Environmental Health, Neuherberg, Germany

**Keywords:** Genetics, Epidemiology, Joint development, Joint shape, Osteoarthritis

## Abstract

Congruent, low-friction relative movement between the articulating elements of a synovial joint is an essential pre-requisite for sustained, efficient, function. Where disorders of joint formation or maintenance exist, mechanical overloading and osteoarthritis (OA) follow. The heritable component of OA accounts for ~ 50% of susceptible risk. Although almost 100 genetic risk loci for OA have now been identified, and the epidemiological relationship between joint development, joint shape and osteoarthritis is well established, we still have only a limited understanding of the contribution that genetic variation makes to joint shape and how this modulates OA risk. In this article, a brief overview of synovial joint development and its genetic regulation is followed by a review of current knowledge on the genetic epidemiology of established joint shape disorders and common shape variation. A summary of current genetic epidemiology of OA is also given, together with current evidence on the genetic overlap between shape variation and OA. Finally, the established genetic risk loci for both joint shape and osteoarthritis are discussed.

## Introduction

Osteoarthritis (OA) is a disorder involving movable joints characterized by cell stress and extracellular matrix damage leading to cartilage degradation, bone remodelling, osteophyte formation, joint inflammation and loss of normal joint function that manifests clinically with pain, deformity, and disability https://www.oarsi.org/education/oarsi-resources/oarsi-white-paper-oa-serious-disease. Approximately, 240 million people (3.3% of the world’s population) live with OA [1]. Between 1990 and 2013 osteoarthritis was responsible for a 75% increase in years living with disability to 13 million [2], behind only diabetes (135%) and dementia (84%) in increasing prevalence. This reduced physical activity results in an increased all-cause mortality for OA sufferers versus the general population (standardised mortality ratio 1.55, 95% confidence interval 1.41–1.70) [3], largely attributable to an excess cardiovascular risk. In 2003, OA accounted for 1.2% of United States domestic product ($128billion, direct costs $80Billion and loss of earnings $47Billion) [4]. By 2013, this figure had increased to $304Billion [5].

The epidemiological risk factors for OA are well-established and include older age, female sex, obesity, joint injury, bone morphology and family history. The heritability of OA has been estimated in studies of monozygotic versus dizygotic twins to range between 40% (knee) and 60% (hip) [6, 7], and follows a non-Mendelian pattern consistent with the common, complex nature of the disease. The interaction between environmental and heritable risk factors for OA also differs with age, between men and women, and with body mass index [8]. In recent years it has become more widely appreciated that underlying joint shape is a strong risk factor for OA. This realisation has led to an explosion in the use of surgical interventions designed to restore the joint to a more anatomically normal shape and improve mechanical symptoms [9–11]. Only time will establish whether such interventions alter the natural history of OA. In this article, we provide a brief overview of the morphology and development of synovial joints, review the genetic epidemiology of joint shape as it relates to OA genetic epidemiology, and consider to what extent this heritable component is shared.

## What is a Synovial Joint?

Synovial joints, characterised by a fluid-filled synovial space between the bones, are the most flexible type of joint in the body, allowing movement between the bone ends in up to 6 degrees of freedom and across an arc of movement of up to 140 degrees. Synovial joints are the most common type of joint, and allow great facility in both the range and type of movement from repetitive, weight-bearing activity through to intricate, fine motor functions. Such freedom of movement and resilience requires exquisite design. All synovial joints comprise subchondral bone that is lined by hyaline cartilage and enclosed by a strong fibrous capsule that is lined by synovial tissue. The outer fibrous capsule is reinforced by ligaments that act as primary stabilisers of the joint. The joint cavity is filled with lubricating synovial fluid that is synthesised by the synovial membrane and hyaline cartilage, allowing low-friction motion between the joint elements. The joint cavity may also contain fibrocartilage extensions of the capsule that function to spread load more evenly within the joint and act as a secondary stabiliser (knee menisci and hip labrum). Further stabilisation of the joint is provided by accessory ligaments that may be separate to, or fused with, the capsule.

Synovial joints perform a variety of movements, necessitating variation on the same basic design. These include pivot joints that allow rotation (upper cervical vertebra), hinge joints that allow movement in only 1 plane but can allow transfer of large forces to create great power (knee, elbow), saddle joints that allow fine multidirectional movement (small joints of the hand), plane joints that allow limited sliding motion whilst resisting large forces (midfoot), condyloid joints that allow complex composite movement (wrist), and ball and socket joints that can rotate and flex almost freely in any direction whilst maintaining great stability (shoulder, hip).

## An Overview of Synovial Joint Development

**Fig. 1:**
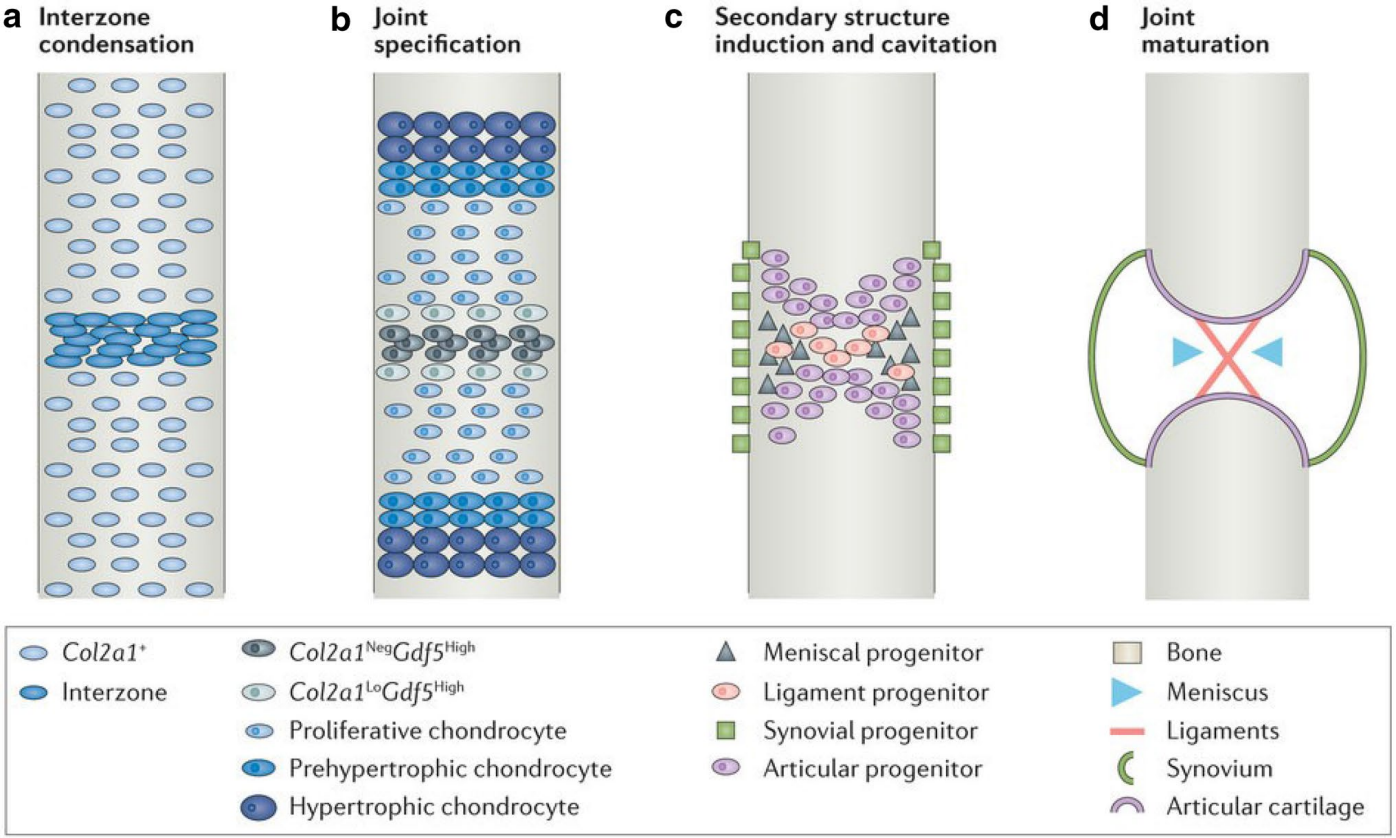
Synovial joint development in the mouse. Longitudinal views depicting key steps in the formation of the knee joint. **a** The first sign of a presumptive joint is a condensation of Col2 + limb bud progenitors at the presumptive joint site; **b** Joint specification is marked by induction of Gdf5 in the interzone and downregulation of Col2a1; **c** A joint space is formed by cavitation after progenitors for a variety of secondary joint structures are specified from the Gdf5 + progenitor pool; **d** Maturation of the synovial joint of the knee occurs during development and early postnatal life. Reproduced from Salazar et al. [165], with permission

Despite the heterogeneity of synovial joint morphology, common signalling pathways underpin their development. In this section, we give a brief overview of the process, although dedicated reviews of this topic can be found elsewhere [12, 13]. Bones and joints develop in mesoderm, comprising mesenchymal cells that have the ability to differentiate into fibroblasts, chondroblasts or osteoblasts and the blood vessels and connective tissues of the limb. Synovial joint formation begins at approximately week 7 of embryogenesis as interzonal mesenchyme between the developing bones [14]. Figure 1 shows joint development from mouse data, but the same stages are followed in humans. The outer layers of the interzone form the long bone epiphyses through endochondral ossification [15], whilst the inner layer differentiates into the articular surfaces. Removal of the interzonal mesenchyme during embryonic development results in fusion of the adjacent developing bones [16].

At the molecular level, the appearance of the interzone is accompanied by a decrease in type II collagen expression [17], and an increase in growth differentiating factor 5 (GDF5) and bone morphogenetic protein 2 (BMP2) expression. GDF5, a member of the transforming growth factor beta superfamily [18], is a key early marker of interzone development and segmentation of skeletal elements [19, 20]. *Gdf5* knockout results in failure of synovial joint development in mice [20]. Whilst GDF5 is an essential requirement for joint development, it is not specific to synovial joints, and its over-expression also results in failure of joint formation, increase in size of the skeletal elements and over-proliferation of epiphyseal cartilage [21].

Interzone expression gradients of BMPs and their inhibitors Noggin and Chordin also regulate appropriate synovial joint development [22, 23]. Relative BMP over-expression results in an epiphyseal cartilage phenotype and joint fusion, rather than in hyaline cartilage and normal joint development. Synovial joint development is also critically dependent upon wingless (wnt) 4, wnt9a, and wnt 16 signalling within the interzone [24]. Whilst wnt signalling is not a requirement for joint initiation, it is a requirement for proper development, including the formation of hyaline articular cartilage. *Wnt4* and *wnt9a* knockout does not inhibit joint patterning but results in subsequent fusion [24, 25]. The molecular regulation of early joint patterning is not limited to the interzone. Indian hedgehog (IHH), expressed at the growth plate during long bone formation, also modulates developing joint architecture and *Ihh* knockout is also characterised by joint fusion [26, 27].

Interzone formation is followed by a process of joint cavitation that involves limited cell death [28], alterations in fibrillar collagen from type I to type II [17], and differential growth of the joint elements as the clefts join to form the central synovial cavity, lined by the developing synovium and joint capsule [29]. The articular cartilage is formed by a layer of cells at the end of the epiphyseal growth plate [30]. Peripherally, the interzone mesenchyme gives rise to the fibrous joint capsule and supporting ligaments of the joint. Where the mesenchyme lines the capsule and articular surfaces, the mesenchymal cells form the synovial membrane. These cells subsequently disappear from the articular cartilage surface, probably as a result of joint movement.

**Fig. 2:**
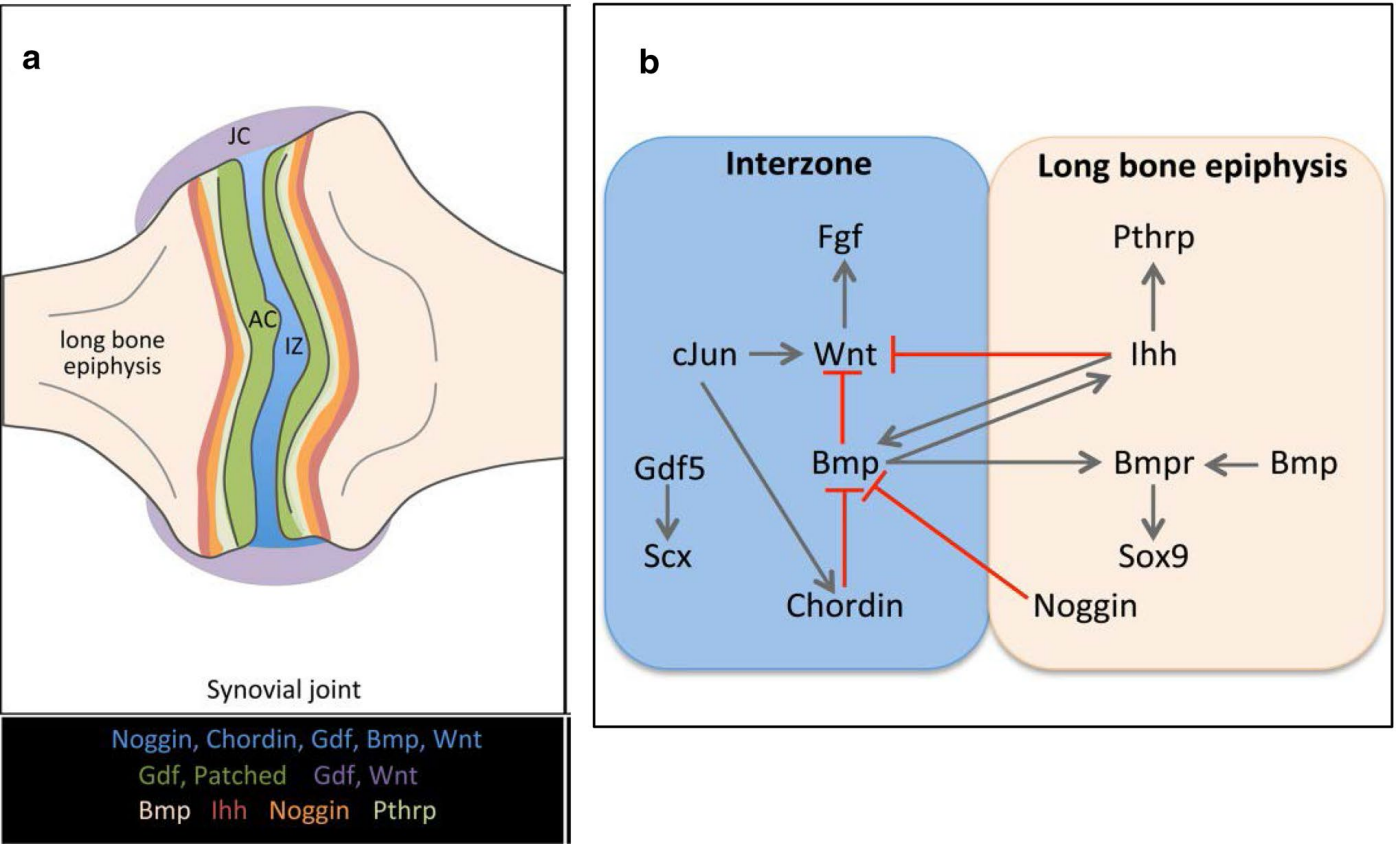
Spatial expression patterns (**a**) and principal signaling pathways (**b**) in synovial joint development. The expression domains of critical signalling pathway components are regionally restricted during development of synovial joints. *AC* articular cartilage, *IZ* interzone, *JC* joint capsule. Reproduced from Salva et al. [12], with permission

The process of joint cavitation is thought to occur largely through mechanically induced changes in the extracellular matrix [30, 31]. Although interzone formation occurs in the absence of movement, skeletal muscle activity is required for both cavity formation and subsequent morphogenesis [14, 32–34]. During this period there is also an increase in expression of CD44 and increased hyaluronan synthesis that facilitates tissue separation and formation of a functional cavity [35], which is also thought to be under mechanical regulation [36]. Studies show that muscle imbalance during development results in abnormally shaped joints [37–39]. There is also evidence that some signalling mechanisms may be joint-specific [20, 40]. An overview of the signalling networks involved in synovial joint development and their spatial colocation is shown in Fig. 2 (adapted from [12]). Joint growth and modelling continues beyond embryogenesis and throughout postnatal growth to skeletal maturity. This growth is regulated by a complex interplay of local molecular mechanisms that are, in turn, also regulated at the long-range level [41]. These processes are reviewed in detail elsewhere, [13, 42] but where perturbed, joint shape and surface congruity abnormalities may result that can give rise to secondary OA.

In the following sections, we consider several common complex conditions in which joint development is affected pre- or post-natally. The evidence base for these conditions comes from the epidemiological literature that uses its own lexicon of terms. For the purposes of this review, we use the term “risk factors” to mean variables that affect an individual’s chance of developing a disease. We use the term “susceptibility” to mean the individual’s overall chance of developing the disease. Note that the associations between individual risk factors and overall disease susceptibility may be causal (in which there is a direct mechanistic link), or represent a marker for the disease for which the mechanism of the link is unclear but the two are clearly associated (such as through a third factor). The terms variant and single nucleotide polymorphism (SNP) are used synonymously. We use the term “common variant” to describe risk alleles with a frequency of > 5% (or 0.05). Low-frequency variants have an allele frequency of between 1 and 5% (0.01 to 0.05), and rare variants have a frequency of < 1% (or 0.01).

## How Does Genetic Variation Influence Joint Architecture?

As outlined above, it is clear that a complex interplay of molecular signalling and mechanical events are required for normal synovial joint development. Whilst loss of function mutations within genes critical to synovial joint morphogenesis lead to generalised musculoskeletal skeletal abnormalities that may be incompatible with life, polymorphisms that arise in viable general populations also give rise to variations in joint shape that are heritable. The former group comprises a multitude of genetic mutations that provide critical insights into the functioning of individual genes required for normal musculoskeletal development. Here, we focus on common, complex disorders that arise within general populations and associate with the development of OA. These disorders may be broadly classified into those that are captured within established disease definitions, and those that represent statistical shape variation within populations.

The hip is the most common site for established pathological variation in joint shape. Recognised disorders of hip joint morphology during growth include developmental dysplasia of the hip (DDH), Perthes’ disease, slipped capital femoral epiphysis (SCFE) and femoroacetabular impingement (FAI). These diseases describe discrete pathological entities that are associated with specific clinical shape phenotypes.

### Developmental Dysplasia of the Hip

**Fig. 3:**
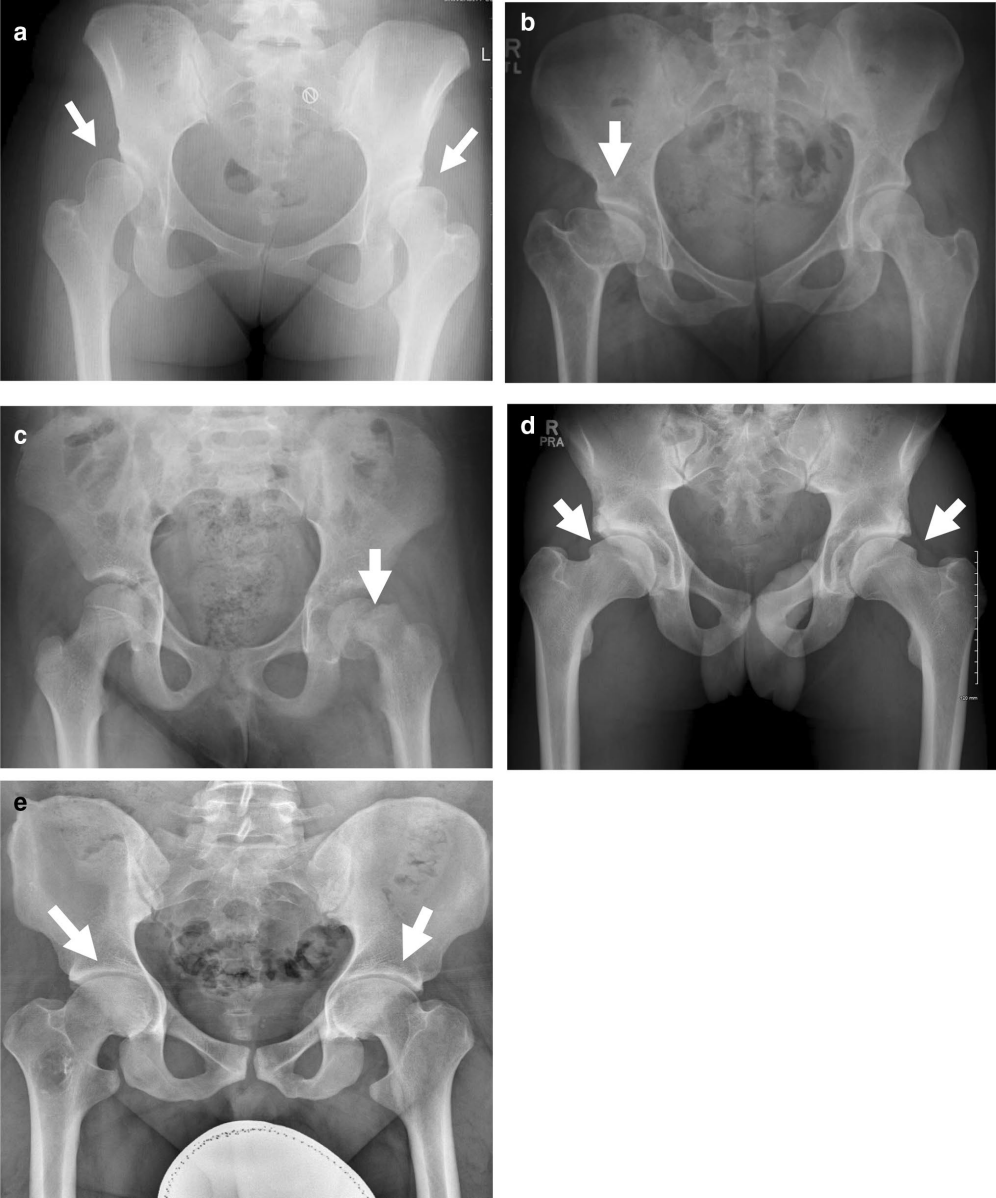
Disorders of the growing hip joint. **a** Bilateral hip dysplasia in a skeletally mature individual. The acetabuli are typically shallow and steep, and there is extrusion of the femoral heads with evidence of decreased lateral coverage (arrows); **b** Perthes’ disease of the right hip in a skeletally mature individual. The right femoral head is broad and flattened and the corresponding acetabululm is similarly shaped (arrow). **c** Slipped capital femoral epiphysis of the left hip in an adolescent male. The epiphysis has slipped posteromedially from the physis (arrow); **d** Bilateral cam lesions in a skeletally maturing male, arrows show lateral head-neck junction prominence; **e** Bilateral pincer lesions in a skeletally mature male, arrows show over-coverage of femoral head

DDH, the most common skeletal dysplasia, is characterised by abnormal development of the hip joint and presents with varying severity from mild uncovering of the femoral head to complete dislocation of the hip joint and acetabular aplasia (Fig. 3a) [43]. DDH has an incidence that ranges from 0.06 per 1000 live births in individuals of African ancestry to 76.1 per 1000 live births in Native Americans, and with an incidence in the UK European population of 3.6 per 1000 [44]. The aetiology of DDH is complex, involving both environmental and genetic risk factors. Known associations include female sex, first-born, breech presentation, and family history [44].

Family studies have demonstrated that heritable factors contribute between 50 and 85% of the total DDH susceptibility [45–47]. Stevenson et al. [48], in a familial aggregation study of 1649 distinct individuals from Utah, USA, with a DDH case: control ratio of 1:10, found a recurrent risk of 12 in first-degree relatives of those with DDH versus controls. Li et al. [46], in a case–control familial study of Chinese individuals with DDH, reported a heritability of 84%, and a sibling recurrent risk of probands that was tenfold that of the siblings of controls. Heritability may also be estimated at the population level amongst unrelated individuals using genome-wide association study (GWAS) analysis approaches. Hatzikotoulas et al. [49], using genetic complex trait analysis [50] across 770 DDH cases and 3,364 controls in a discovery scan of 257,000 directly typed genetic variants with a minor allele frequency of > 0.01, found that common variants explained 55% of the liability-scale heritability, and was equally distributed across chromosomes.

Genome-wide linkage analysis (GWLA) has identified several chromosomal regions segregating within large DDH pedigrees. Feldman et al. [51], in a GWLA on an 18-member multigeneration family, the proband of which was severely affected by DDH, identified a 4 Mb region on chromosome 17q21. Candidate genes coded in this region are the *HOXB* cluster of homeobox genes, *COL1A1* and *DLX3*. A Japanese GWLA of a 4-generation family containing eight patients with familial osteoarthritis of the hip associated with acetabular dysplasia suggested linkage at 13q22 [52]. A GWLA of a large South African family of European origin with Beukes hip dysplasia, an autosomal dominant disorder of variable penetrance that is characterised by bilateral dysmorphism of the proximal femur, mapped the causal gene to a 3.34 Mb region at 4q35 [53]. Several candidate gene studies have also suggested associations between genetic variants and DDH, including *GDF5*, *TBX4*, *ASPN*, and *IL6*, although only *GDF5* has been independently replicated and reached genome-wide statistical significance levels (Table 1).

**Table 1:** Summary of the published genetic associations with DDH

Study reference	Gene/loci	Chromosomal location	Study design	SNP variant	Phenotype	Study population(s)
Feldman et al. (2010) [51]	17q21.31–17q22	17q21.31–17q22	GWLA	–	DDH	American and Chinese
Mabuchi et al. (2006) [52]	13q22	13q22	GWLA	–	DDH	Japanese
Watson et al. (2015) [53]	*UFSP*	4q35	GWLA and exome sequencing	c.868T > C	Beukes hip dysplasia	South African
Feldman et al. (2013) [153]	*CX3CR1*	3p22.2	GWLA and exome sequencing	rs3732378	DDH	American
Basit et al. (2017) [154]	*HSPG2*	p. Ala1110Ser	GWLA and exome sequencing	c.3328G > T	DDH	Saudi Arabian
Basit et al. (2017) [154]	*ATP2B4*	p. Arg755Gln	GWLA and exome sequencing	c.2264G > A	DDH	Saudi Arabian
Dai et al. (2008) [155], and Hatzikotoulas et al. (2018) [49]	*GDF5*	20q11.22	CGAS GWAS and subsequent replication	rs143383, rs143384	CDH DDH	Chinese females United Kingdom
Wang et al. (2010) [156]	*TBX4*	17q23.2	CGAS	rs374448	DDH	Chinese
Shi et al. (2011) [157]	*ASPN*	9q22.31	CGAS	D-repeat polymorphism of ASPN	DDH	Chinese
Cengic et al. (2015) [158]	*IL6*	7p15.3	CGAS	rs1800796	DDH	Croatian
Cengic et al. (2015) [158]	*TGFB1*	19q13.2	CGAS	rs1800470	DDH	Croatian
Jia et al. (2012) [159]	*PAPPA2*	20q11.22	CGAS	rs726252	DDH	Chinese
Hao et al. (2014) [160]	*HOXB9*	17q21.32	CGAS	rs2303486	DDH	Chinese
Tian et al. (2012) [161]	*HOXD9*	2q31.1	CGAS	rs711819	DDH	Chinese females
Liu et al. (2014) [162]	*DKK1*	10q21.1	CGAS	rs1569198	DDH	Chinese
Zhao et al. (2013) [163]	*COL1A1*	17q21.33	CGAS	rs113647555	DDH	Chinese females
Sun et al. (2015) [164]	*UQCC*	20q11.22	GWAS	rs6060373	DDH	Chinese

*GWLA* genome-wide linkage analysis, *CGAS* candidate gene association study, *GWAS* genome-wide association study

Genome-wide association analysis enables a hypothesis-free approach to interrogating the entire human genome for potential associations with a disease. In a GWAS of 770 DDH cases and 3364 population-based controls, Hatzikotoulas et al. [49] identified eleven correlated variants at genome-wide significance (*P* < 5.0 × 10^−8^) residing within the 5′untranslated region of *GDF5* (20q11.22). Independent signals were replicated in three DDH cohorts of UK European ancestry, totalling 1129 cases and 4652 controls. Following meta-analysis, rs143384 in *GDF5* (OR [95% CI] 1.44 [1.34–1.56], *P* = 3.55 × 10^−22^) was robustly associated with DDH, reaching genome-wide significance in both the discovery and the replication cohorts. Gene-based association analysis in this cohort also implicated variation within *UQCC1*, *MMP24*, and *RETSAT* at *P* < 5 × 10^–8^. *UQCC1* lies adjacent to *GDF5* and encodes a trans-membrane protein ubiquinol-cytochrome-c reductase complex chaperone. *UQCC* is expressed in differentiating chondrocytes and regulates growth control [54, 55]. Variants in this gene are also associated with bone size [56], height [57] and hip axis length [58]. *MMP24* encodes a member of the peptidase M10 family of matrix metalloproteinases that are involved in the breakdown of extracellular matrix in embryonic development and tissue remodelling [59]. Sequence variants within *MMP24* have also been associated with childhood height [60, 61]. *RETSAT* codes for retinol saturase, an enzyme centrally involved in the metabolism of vitamin A [62]. Retinoic acid signalling is essential for normal limb bud development, including bone and cartilage formation [63].

### Perthes’ Disease

The term Perthes’ disease describes idiopathic osteonecrosis of the femoral head in children. Depending upon the age of onset and the capacity of the developing hip to remodel, the disease results in femoral head and acetabular shape abnormalities of varying severity (Fig. 3b). Perthes’ disease varies greatly in incidence between geographic areas and ethnic groups, but in Western European populations the prevalence is typically between 5 and 15 per 100,000 children under the age of 15 years [64]. The condition is thought to have a multifactorial inheritance pattern, with a sibling and offspring recurrent risk of 2.6% in the UK, based upon the family history of 412 index cases [65]. In a recent study using the Danish Twin Register, Metcalfe et al. examined 81 twin pairs [66] and identified a familial clustering but no genetic component, with an overall proband concordance of 0.09. Although several small case control association studies of the disease have been conducted, including a meta-analysis of hypercoagulability genetic polymorphisms totalling 824 cases and 2033 controls [67], only variation within Factor V Leiden thrombophilia (an inherited disorder of blood clotting) was identified as a possible risk locus (pooled OR 3.10 [95%CI 1.68–5.72]) [67]. Thus, although geographic and familial clustering is clear in Perthes’ disease, the genetic component appears to be small. However, this may be due to the limited sample size and hence power of studies to date.

### Slipped Capital Femoral Epiphysis

Slipped capital femoral epiphysis (SCFE) is a disorder of the hip that is characterised by displacement of the capital femoral epiphysis from the metaphysis through the physis, and results in a characteristic shape abnormality of the hip joint (Fig. 3c). It is a disorder of the adolescent developing hip, is more common in males, and has an incidence that is similar to that of Perthes’ disease [68]. The condition is thought to have an endocrine aetiology and can cluster in families [69, 70], but to date, powered studies to determine any heritable component have not been conducted.

### Femoroacetabular Impingement

The term FAI describes morphological abnormalities of the femoral head–neck junction, acetabulum, or both, resulting in abnormal contact between the proximal femur and the pelvic acetabulum at the hip joint [71]. Two basic pathologies are described. In “Cam” morphology, a non-spherical extension of the femoral head–neck junction (usually anterolateral) causes abutment into the adjacent acetabular cavity [72] (Fig. 3d). In “Pincer” morphology, local or global over-coverage of the acetabulum results in linear impact of the acetabular rim against the head-neck junction [73] (Fig. 3e). The two deformities may also coexist in “Combined” lesions. The FAI hip shape abnormality develops during the adolescent phase of skeletal growth before closure of the growth plate and has been associated with higher levels of sporting activity [74], although a causal link to exercise remains unclear. Asymptomatic FAI is highly prevalent (estimated at up to 75%) in populations of European descent (with cam lesions being more common in males) but is rare in Asian populations [75–78].

The heritable basis of FAI as a distinct hip shape abnormality has not yet been studied in depth. This is perhaps unsurprising, as its development can only be definitively determined at closure of growth plate [79], and there are few clinical reasons to image the hip in asymptomatic young adults. However, small-scale analyses do exist. Pollard et al. compared 96 siblings (mean age 38 years) of 64 patients (mean age 36) treated for FAI with a spouse control group of 77 individuals (mean age 42) and found a relative risk of cam morphology of 2.8 (95%CI 1.8–4.2) and of pincer morphology of 2.0 (1.3–3.0). Pollard et al. also examined familial associations in ‘sibkids’ [80], a cohort with a hereditary predisposition to hip osteoarthritis, and estimated an odds ratio for cam morphology of 2.1 (1.3–3.5) versus spouse controls but no differences in pincer morphology or dysplasia. However, subjects were examined in mid-life when degenerative change is prevalent and a possible common environment underpinning of the associations were not examined. To date, no well-powered genome-wide or candidate variant analyses have been published that examine the relationship between specific genetic risk factors and conventional radiographic indices of FAI.

### Population Level Variation in Joint Shape

At the population level, computer vision methods have been applied to examine joint shape variation, although these phenotypes do not directly map to the clinical diagnoses of FAI or DDH. Statistical shape analysis describes the shape of a deformable object by applying principal component analysis to a set of landmark points, and assumes each shape is a deformed version of a reference shape. Statistical shape models (SSM) may be applied to both 2-dimensional (2-D) and 3-D medical images [81]. SSMs have been used to describe the principal sources of population variation in shape at both the hip and the knee [82–85], and to describe those associated with trochlear dysplasia [86] and acute anterior cruciate ligament injury [87]. In the setting of FAI, SSMs have also been used to compare cam lesion deviation from the population reference femoral head-neck shape in pre-operative planning for surgical intervention [88].

The epidemiology of bone geometry has been of interest in the exploration of the association between hip shape and osteoporotic fracture risk. In meta-analyses of overlapping patient cohorts, Baird et al. [89], and Hsu et al. [90] examined genome-wide genotyped datasets in patients with DXA scans of the hip to identify relationships with proximal femoral SSMs and conventional measures of hip geometry, respectively. Baird et al. used SSMs to examine shape-genotype associations in ~ 16,000 individuals across ~ 7 million SNPs, identifying eight independent genome-wide significant variants (Bonferroni-corrected *P* < 5 × 10^–9^) that were associated with three modes of joint shape variation [89]. Seven of the variants were located within 200 kb of genes involved in endochondral ossification (*SOX9*, *PTHrP*, *RUNX1*, *NKX3-2*, *FGFR4*, *DICER1*, and *HHIP*), suggesting possible association with bone formation and by extension, joint shape. Hsu et al. examined conventional DXA-derived hip structural analysis in 18,719 men and women. Using linkage disequilibrium (LD) score regression [91], they found that nominally-associating variants explained 12%, 13%, 18%, and 22% of the heritable variation in neck-shaft angle, femoral neck length, neck section modulus, and narrowest neck width, respectively. Following replication in independent cohorts, independent variants near *IRIX/ADAMTS16*, *LRP5/PPP6R32/GAL*, *CCDC91*, *FGFR4*, *NSD1*, and *RAB24* met genome-wide significance.

In a GWAS of bone area as a proxy for bone size quantitated by DXA, Styrkarsdottir et al. identified 12 loci that are associated with hip or vertebral size [92], examining 33.4 million sequence variants in Icelandic subjects across 28,954 hip scans and 29,059 lumbar spine scans. They identified rs143384 (20q11.22) in the *GDF5* 5′UTR as a locus for total hip (*β* = 0.071, *P* = 2.2 × 10^–22^) and trochanteric area (*β* = 0.071, *P* = 1.1 × 10^–18^). This locus had previously been robustly associated with DDH in the GWAS by Hatzikotoulas et al. [49]. They also identified two other hip area loci, 17q24.3 in an intergenic region near *SOX9* (intertrochanteric area *β* = 0.072, *P* = 6.2 × 10^–18^) and 4q31.21 in the 5′UTR of *HHIP* (femoral neck area *β* = 0.054, *P* = 8.4 × 10^–14^), that had previously been associated with hip shape SSMs in the Baird GWAS meta-analysis [89].

## Joint Shape is an Important Risk Factor for OA

### Conventional Measures of Joint Shape and OA Susceptibility

**Fig. 4:**
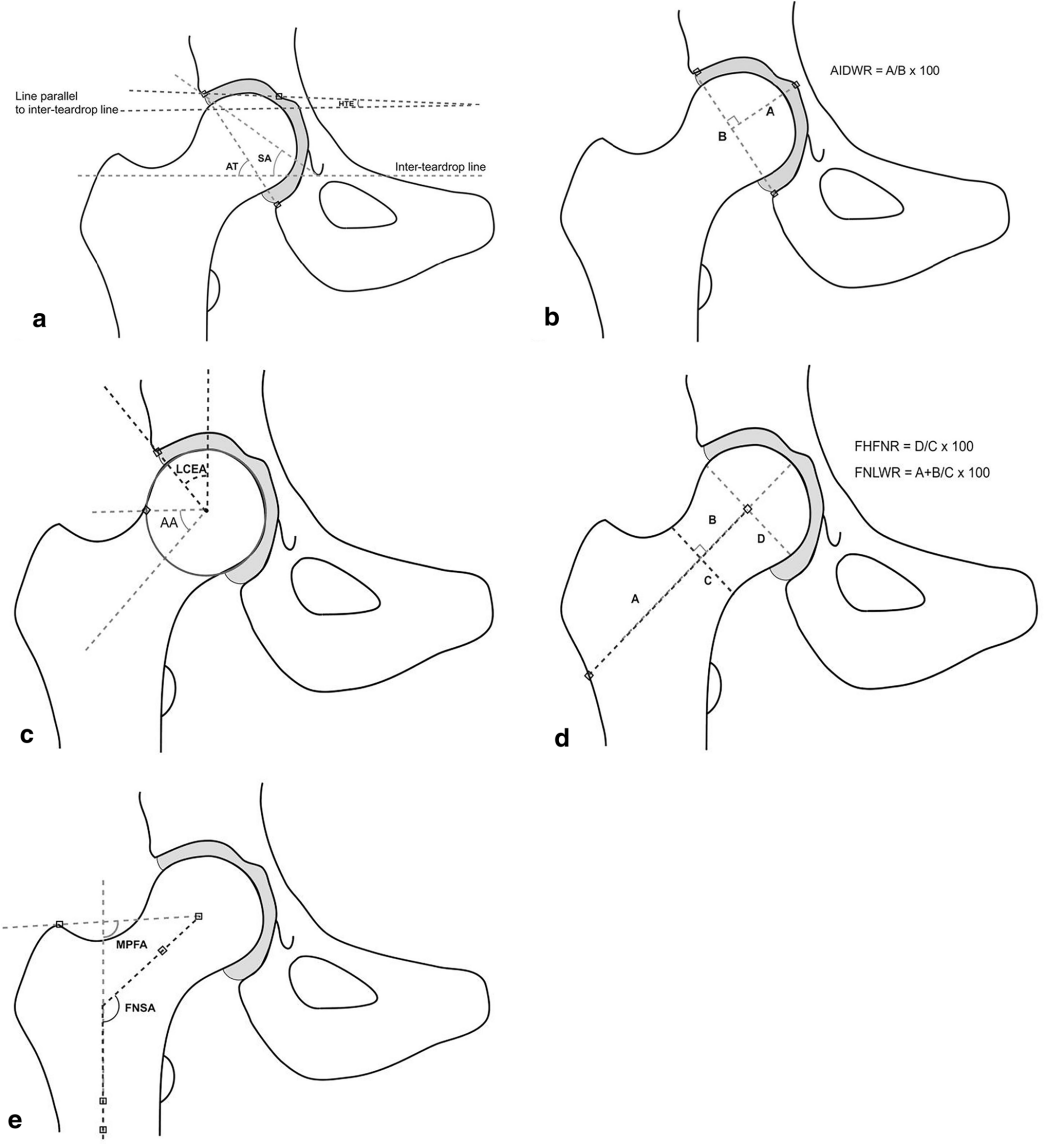
Acetabular and proximal femoral morphological parameters associated with OA risk. **a** *HTE* horizontal toit externe, *SA* Sharp’s angle, and *AT* acetabular tilt; **b** *AIDWR* acetabular index of depth to width ratio; **c** *LCEA* lateral centre-edge angle and *AA* alpha angle; **d** *FHFNR* femoral head to femoral neck ratio and *FNLWR* femoral neck length to width ratio; **e** *MPFA* modified proximal femoral angle and *FNSA* femoral neck-shaft angle

**Fig. 5:**
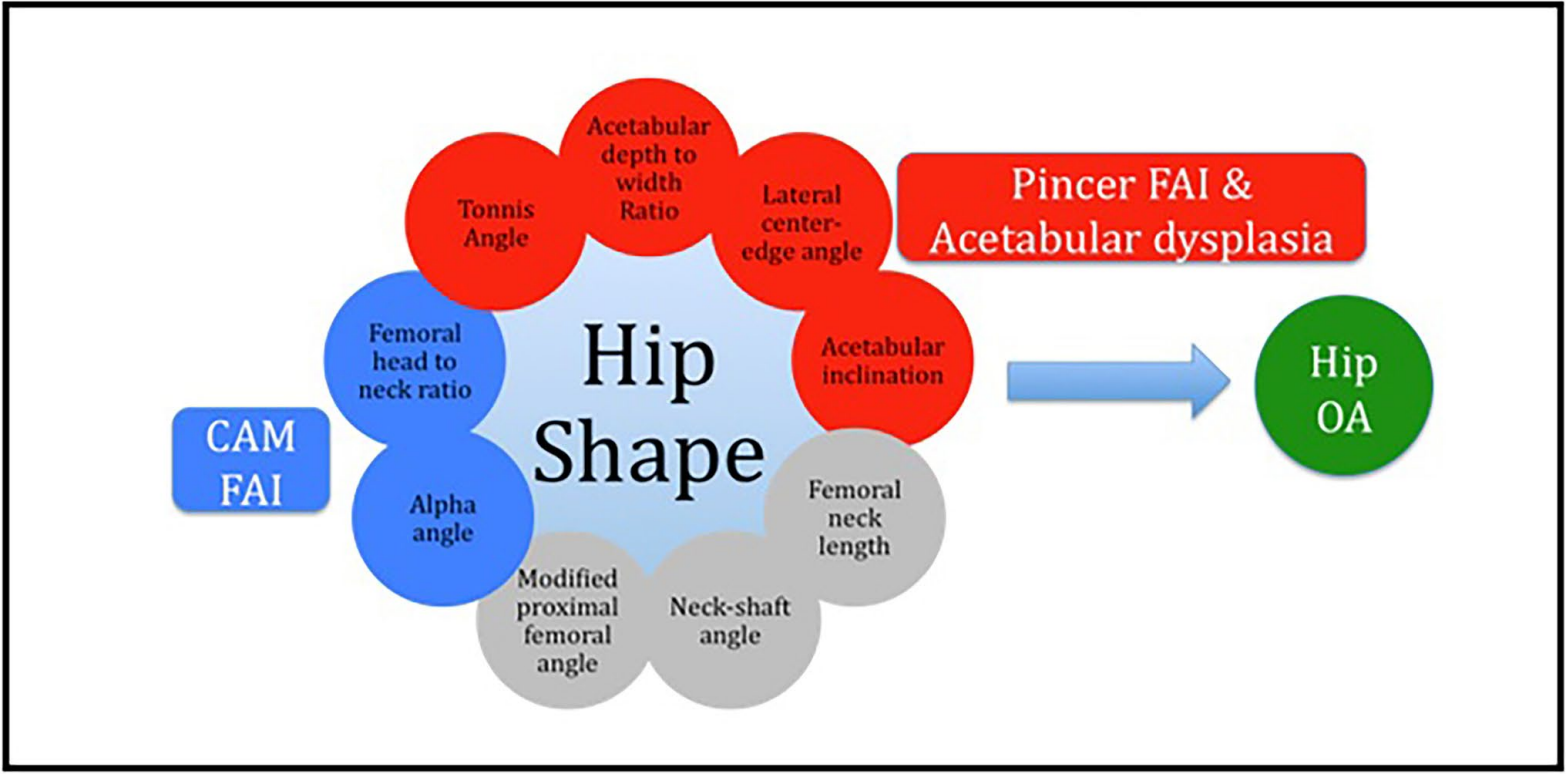
The biomechanical consequences of non-normal hip shape. Illustration shows relationship between out of range hip shape parameters and the development of osteoarthritis

It has long been recognised that pathological developmental abnormalities of the hip joint such as DDH, Perthes’ disease, and SCFE commonly result in osteoarthritis and hip replacement [49, 93–95]. For example, individuals with mild DDH are 10 times more likely to develop OA compared with DDH-free controls [96]. It has only been more recently appreciated that subtle variations in joint shape also predispose to degenerative change. Such variations in joint shape are commonly measured on plain radiographs in clinical practice to describe joint geometry (see Fig. 4 for common indices and their definitions). Observations by Murray [97], and later by Solomon [98] and Harris [99], estimate that over 90% of individuals with idiopathic hip OA exhibit subtle variations in acetabular and proximal femoral shape that precede the degenerative process (Fig. 5). These deformities are highly prevalent in European populations and can result in a focal mechanical overload of articular cartilage, leading to subsequent OA [72, 73, 100]. Doherty et al. [101], in a study of plain anteroposterior radiographs of the hip in 965 OA cases and 1111 controls without radiographic OA, found the presence of cam morphology in at least 1 hip in 18% of cases versus 4% of controls (OR 6.95; 95%CI 4.64–10.41). An abnormal femoral head-to-neck ratio was present in of 24% in cases versus 4% in controls (OR 12.01; 8.05–18.15). As the femoral head-to-neck ratio decreased, the presence of hip OA also rose.

Demonstration that the converse is also true was made by Kim in 1989, reporting a high incidence of hip joint congruity and low incidence of both DDH and of OA in the Korean population [102]. Dudda et al. [103], compared morphological features associated with FAI in females from a Chinese cohort (Beijing OA Study) versus white women from the United States participating in the Study of Osteoporotic Fractures. They found low lateral centre-edge angles suggesting dysplasia (< 20°) more often in the Chinese (22% of hips versus 7% in whites, *p* = 0.005), whilst low mean impingement angles (83.6° versus 87.0°, *p* = 0.03) and lateral centre-edge angles suggestive of impingement (23% versus 11%, *p* = 0.008) were more common in women of European descent. More recently, Edwards et al. examined hip plain radiographic morphological characteristics in individuals of European descent, African Americans and Chinese men and women with no radiographic hip OA [104]. They found that the Chinese group had more shallow and narrow acetabular sockets, reduced femoral head coverage, smaller femoral head diameter, and a lesser femoral neck-shaft angle versus the other ethnic groups. Taken together, these findings are consistent with the epidemiological observation that idiopathic OA is more prevalent in Europeans [105], whilst in Asian populations hip OA is more commonly associated with a DDH morphology.

Deformities associated with cam-type FAI and subtle DDH morphology are also independent predictors of incident radiographic OA and progression to hip replacement in longitudinal studies. Nicholls et al. [106], found that cam morphology, identified by an alpha angle of greater than 65°, had an increased risk of radiographic OA and hip replacement over 2 decades [107], with each degree increase in alpha angle conferring a 5% and 3% increase in risk respectively. Agricola et al. found similar relationships between increasing alpha angle (> cam morphology) and OA progression in the CHECK cohort [108]. Thomas et al. [107], found that mild DDH increased the risk of radiographic OA development and hip replacement over 2 decades, with each degree reduction in lateral centre edge angle below 28° associated with a 14% and 21% increase in risk of OA and hip replacement, respectively [107]. However, no clear associations have been identified between isolated pincer-type FAI and future OA risk [106–109].

### Statistical Models of Joint Shape and OA Susceptibility

SSMs capture information that is predictive of OA and that is not present in predefined radiographic geometric measures, and vice versa. When combined, they add to the predictive value for incident OA above clinical risk factors. Waarsing et al. [110], examined DXA images of the hip in 218 men and women with established hip OA followed over 2 years. They demonstrated that various shape and texture modes correlated with clinical pain and function scores where plain radiographic measures (Kellgren-Lawrence (KL) score and minimum joint space width) did not. Castaño-Betancourt et al. [111], used SSMs and predefined geometry parameters derived from baseline plain hip radiographs to predict incident radiographic OA (KL score ≥ 2) over 6.5 years in 688 individuals from the Rotterdam Study. They found that hip geometry alone was of similar predictive value to clinical risk factors of sex, age, and body mass index; and when combined, added 7% to the prediction obtained by clinical risk factors alone (AUC = 0.67 (geometry), 0.66 (clinical), 0.73 (geometry + clinical) [111]. Agricola et al. demonstrated in female participants in the CHECK and Chingford cohorts that baseline (KL < 2) radiograph-derived hip SSMs were also associated with future THR [112].

SSMs are also emerging as a useful phenotyping tool to improve our understanding of the relationship between genotype and joint shape. In the first of such studies, Baker-LePain et al. [113], examined the association of candidate variants within the wnt antagonist *FRZB* with radiograph-derived hip shape SSMs in a nested subsample (European-ancestry women ≥ 65 years; 451 hip OA cases, 601 controls) from the Study of Osteoporotic Fractures. They found no association between genotype and case–control status or with acetabular depth or centre edge angle, but a weak association with mode 2 (amongst 10 modes) in subjects having at least 1 copy of the rs288326 or the rs7775 *FRZB* minor allele (*P* = 0.019 for each test). A subsequent analysis stratified by presence of the rs288326 minor allele showed a weak positive association between the upper quartile of subjects for mode 2 with OA in those carrying the variant allele (*P* = 0.02), which led them to propose that genetic variation within *FRZB* may modulate the effect of hip shape on OA risk.

Subsequently, Lindner et al. examined 41 candidate genetic variants (that had previously been associated with OA or hip morphology) and plain anteroposterior radiographs of the non-OA-affected hip in 929 patients from the arcOGEN cohort [114, 115]. In univariate analysis they identified an association between rs4836732 (within *ASTN2*) and mode 5 of the female SSM (*p* = 0.0016), and between rs6976 (within *GLT8D1*) and mode 7 of the mixed sex SSM (*p* = 0.0003). The multivariate analysis identified association between rs5009270 (near *IFRD1*) and a combination of modes 3, 4, and 9 of the mixed-sex SSM (*p* = 0.0004). In the Baird et al. hip shape GWAS [89], 3 of the genome-wide significant variants that were associated with shape mode variation are also established loci for hip OA, rs4836732 (intronic variant within *ASTN2*), rs10743612 (intergenic variant downstream of *PTHLH*), and rs73197346 (intergenic variant upstream of *RUNX1*).

Fewer studies have examined the relationship between SSMs and knee OA. Haverkamp et al. examined differences in knee shape by SSMs between women with prevalent knee OA versus those without OA in Rotterdam Study participants [116]. They found that the women with knee OA had SSMs that described a broader femur and tibia and also an elevated lateral tibial plateau, findings that are consistent with plain radiographic features in the presence of knee OA. Wise et al. [117], in a study of 304 knees with (KL ≥ 2) versus 304 knees without (KL < 2) incident OA from the Osteoarthritis Initiative found that SSMs of distal femoral and proximal tibial shape derived from plain anteroposterior knee radiographs only weakly and inconsistently modulated the relationship between sex and incident knee OA.

### Bone Shape Remodelling in Response to OA

Care is required when interpreting the results of joint shape and OA susceptibility association analyses, as joint shape changes with OA severity. Gregory et al. [118] used SSMs of the proximal femur derived from plain radiographs of the hip at baseline and again after 6 years in 110 participants in the Rotterdam Study to quantify the deforming effect of OA progression on the proximal femur, and first proposed SSMs as an imaging biomarker of hip OA progression. Similarly, Hunter et al. [119], conducted a nested case–control study of knee MRI data within the Osteoarthritis Initiative and showed that bone area increased and shape changed more over 24 months in OA cases versus controls (OR case/control 1.28–1.71 for area, and 1.22–1.64 for shape per SD change in each variable, respectively).

Such observations make the disentangling of causation from association challenging, particularly where low-resolution modalities such as DXA are used in isolation to define the joint shape and absence of OA at baseline. Further, DXA images present a distorted image of the hip that is magnified in the *x*-axis, because of the fan-beam image acquisition [120]. These limitations are not confined to DXA data, but may impact all SSMs based on 2-D imaging. For example, as OA progresses at the hip, fixed external rotation of the hip occurs that may also give the impression of a change in bone area or femoral neck-shaft angle. Similarly, femoral head osteophytes lead to high alpha angles seen in Cam morphology, and lateral acetabular osteophytes produce pincer morphology. Cross-sectional imaging modalities do not suffer such artefacts to the same extent. Recently, Inamdar et al. [121] conducted a 36-month longitudinal MRI study of variation in proximal femur 3D morphological shape (3D-SSM) and associations with cartilage health in 46 men and women with developing hip osteoarthritis. They showed that 3D-SSM characterising increases in head and neck volume and decreasing femoral neck anteversion were weakly associated with progression of symptoms and MRI-identified cartilage lesions.

Bone remodelling features have also been used to define different phenotypes within established OA into normotrophic, hypertrophic, or atrophic, depending upon the resultant bone shape [122]. These differences in bone remodelling responses may also have genetic correlates that further impact on the observed genetic relationships between joint shape and OA. Panoutsopoulou et al. [123], examined the effect of clinically relevant endophenotyping according to site of maximal joint space narrowing (maxJSN) and bone remodelling response in a stratified GWAS of the arcOGEN dataset, comparing 2118 radiographically-defined hip OA cases and 6500 population-based controls. They found that variation within *LRCH1* was associated with site of maximal joint space narrowing (OR 0.70; 0.61–0.80), whilst variation adjacent to *STT3B* was associated with a hypertrophic pattern of remodelling (OR 1.45; 1.24–1.69). Both associations were completely attenuated in the non-stratified analyses. Further, *STT3B* was over-expressed in OA-affected versus intact human cartilage in an analysis of hypertrophic versus atrophic bone remodelling pattern.

## Evidence from OA Susceptibility Studies

**Fig. 6:**
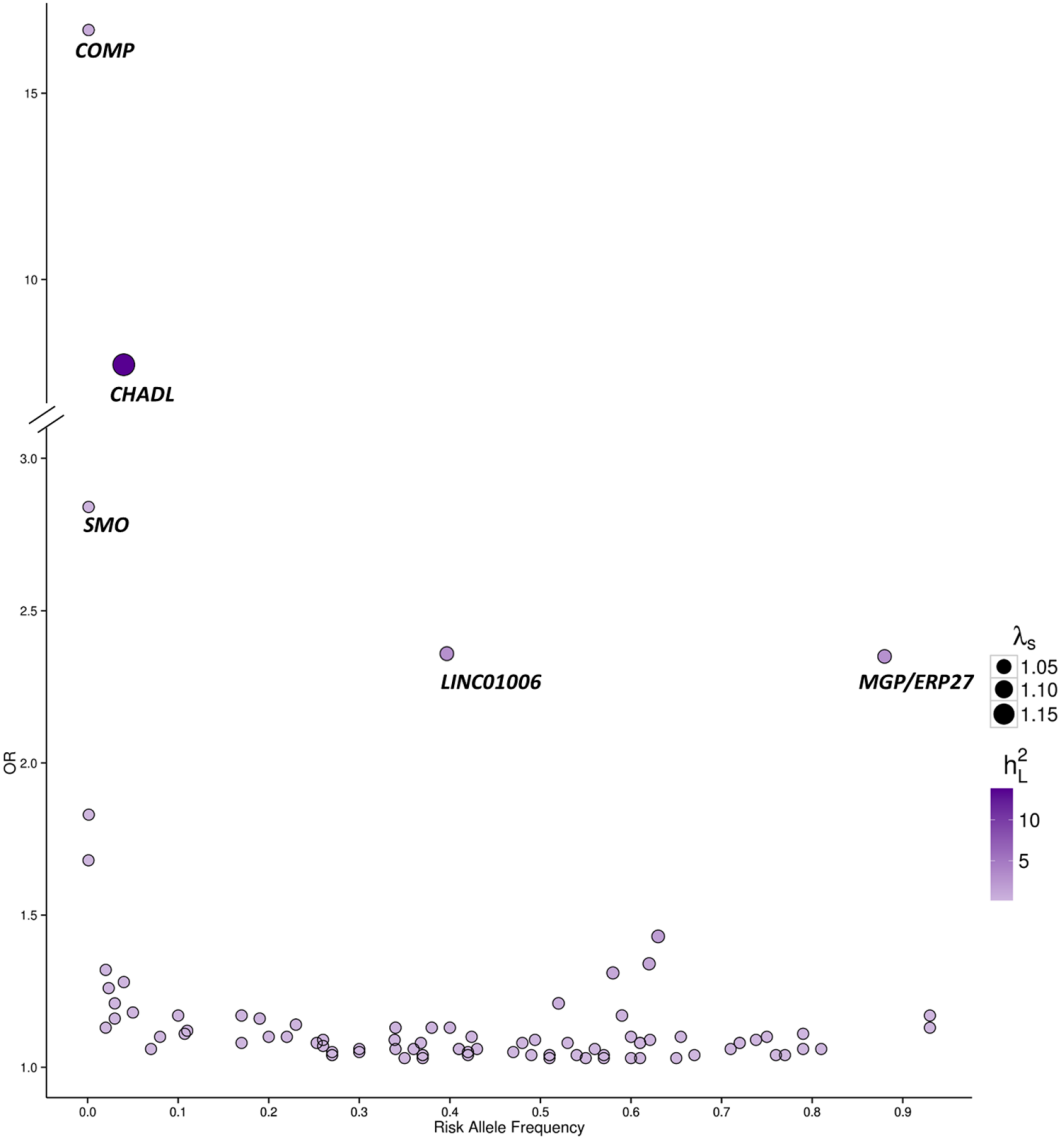
Effect size and risk allele frequency of published osteoarthritis genetic risk loci. Each circle represents a published osteoarthritis risk single-nucleotide variant plotted with its odds ratio (OR; *y* axis) as a function of the risk allele frequency (*x*-axis). The different size and colour of each circle shows the sibling relative risk ratio (λs) and the percentage of variance explained on the liability scale respectively (h^2^L; calculated assuming 13.5% prevalence of OA). Gene annotations are taken from the Ensembl genome browser (human assembly GRCh37). The majority are common variants with small (OR < 2.0) effect sizes, only those with OR > 2.0 are named here

Our understanding of the genetic epidemiology of OA has increased substantially over the last few years with the number of genome-wide scans reported on increasingly large patient cohorts. To date, these efforts have resulted in almost 100 robustly replicating genome-wide significant OA risk loci being identified [124–129]. The vast majority of these risk loci represent common variants with a small to moderate effect size, consistent with the complex and highly polygenic architecture of the disease (Fig. 6). However, large-scale GWAS datasets are not commonly accompanied by radiographic phenotype data upon which to examine relationships with joint shape.

Zengini et al. [124], in a GWAS of the UKBiobank meta-analysed with other cohorts totalling 30,727 cases and 297,191 controls examining 16.5 million variants identified nine novel, robust OA variants. They also conducted a nested substudy of osteoarthritis-related plain radiographic phenotypes in mixed cohorts of smaller size (where radiographic data were available). Of the nine identified variants in the primary analysis, several were associated with the minimum joint space width phenotype, but only a nominal association between an intergenic variant rs116882138 and centre-edge angle (a feature of DDH, *β* = − 1.1388, *P* = 0.03), and no association with alpha angle (a feature of cam morphology), was found. Tachmazidou et al. [125], in the largest GWAS of OA published to date, including 77,052 cases and 378,169 controls in a meta-analysis examining 17.5million variants, identified 64 signals (52 novel) at MAF > 0.01 on top of 34 previously established loci [124, 126, 128, 130–142]. Pathways analysis using MAGMA, PASCAL, and DEPICT identified 64 biological processes, 46 of which are involved in bone, cartilage, or chondrocyte morphogenesis. Although associations with other demographic characteristics and disease states were examined by linkage disequilibrium regression analysis using LDHub [91], specific direct associations with joint shape were not explored.

## What Consistent Signals Arise from Both Joint Shape and OA Studies?

Although many studies have examined the genetics of OA, relatively few have examined the genetics of diseases of joint shape or the relationships between shape, joint shape disorders and OA. Hatzikotoulas et al., used LD regression to estimate the genetic correlation between DDH and hip OA in the UK Biobank hip OA dataset, identifying a positive genetic correlation (rg = 0.58 (s.e. = 0.21), *P* = 0.0047), but did not distinguish between a causal relationship versus shared genetic causes. Baird et al. [143], examined the relationship between 11 genetic loci for hip OA and DXA-derived hip shape in 3111 women participating in the Avon Longitudinal Study of Parents and Children. They identified associations with at least nominal significance between 3 OA risk loci (*KLHDC5/PHTLH* rs10492367, *DOT1L* rs12982744, and *COL11A1* rs4907986) and hip shape. Co-localisation analysis indicated sharing of genetic signals for hip shape and hip OA for the *KLHDC5*/*PTHLH* and *COL11A1* loci. However, the cohort was not screened for the presence of radiographic OA prior to inclusion in the analysis and thus hip shape variation resulting from OA, rather than predisposing to it, cannot be excluded.

Whilst many risk loci that lie adjacent or within genes that have a role in mesenchymal cell function have been associated with subtle statistical variations in joint shape or OA susceptibility, relatively few specific genes have been consistently identified across both the joint shape and the osteoarthritis phenotypes. Some of the key implicated genes are outlined below.

Growth Differentiating Factor 5 (GDF5*)*, a member of the TGFβ signalling family, is a key regulator of joint morphogenesis. Mutations in *GDF5* results in brachydactyly and symphalangism [144]. Variation within *GDF5* has also been widely associated with DDH [49], and also with hip osteoarthritis across multiple populations [124, 130]. Pooled evidence for association from two independent Japanese studies attained genome-wide significance with allelic OR [95%CIs] of 1.79 [1.53–2.09], *p* = 2 × 10^–13^ [130]. This *GDF5* SNP was later associated with knee OA in individuals of European descent in a subsequent meta-analysis across a total of 6861 knee OA cases and 10,103 controls, with allelic OR [95%CIs] of 1.16 [1.10–1.22], *p* = 9.6 × 10^–9^ [145], and in hip and knee OA across 30,727 cases and 297,191 controls [124]. Chen et al. [146] have shown that the *GDF5* locus contains many separate regulatory elements that control expression of the gene at different joint sites, and that these flanking regions are large. Capellini et al. of the same group have also recently described a novel enhancer region GROW1 in an extended downstream regulatory region of *GDF5* [147]. Most recently, the same group has used chondrocyte chromatin datasets to propose a model linking evolutionary genetic variations within the *GDF5-UQCC1* risk locus (rs6060369) that perturb regulatory constraint during knee development with subsequent knee OA in adult mice, and suggest an ortholog in chondrocyte evolution of the modern human knee that affects bone shape [148].

SOX9 is a pivotal transcription factor during joint development and in adulthood, committing mesenchymal progenitors to the chondrocyte lineage, activating cartilage-specific genes and modulating chondrocyte survival. SOX9 deficiency, generally due to spontaneous mutations at the 17q24 locus, causes campomelic dysplasia that is characterised by multiple long bone abnormalities, including brachydactyly and dislocation of the hips [149]. Common variation within *SOX9* is associated with both OA and joint shape variation [89, 92, 125]. *SOX9* lies within a relatively gene-free region on chromosome 17, with large domains (1.9 Mb upstream and 0.5 Mb downstream) that facilitate interaction with *cis*-acting elements causing SOX9-dependent diseases [150].

Parathyroid hormone-like hormone, encoded by *PTHLH*, regulates endochondral bone formation as a downstream signalling pathway to RUNX2, through its receptor’s inhibitory action on chondrocyte development and differentiation and pro-osteoblastic activity [151]. Mutations in *PTHLH* cause brachydactyly and short stature [152], whilst common variation at the *PTHLH* locus is associated with hip shape and OA [128, 143].

Collagen type XI, encoded by *COL11A1*, is an important component of the growing skeleton, but its presence in the adult is mainly confined to articular cartilage and the intervertebral disc. Loss of function mutations In *COL11A1* cause fibrochondrogenesis type 1, a disorder characterised by severe skeletal abnormality and is usually fatal in early life; and Stickler and Marshall syndromes, disorders characterised by less severe bone and joint abnormalities that are non-fatal. Common variation in *COL11A1* is associated with hip shape variation and has been suggestively associated with hip OA, although not at genome-wide significance [114, 143]. The molecular mechanisms of these proposed variants on *COL11A1* expression remain to be clarified.

Astrotactin 2, encoded by *ASTN2*, is most strongly expressed in the brain and plays a role in neuronal migration. *ASTN2* deletion causes autism and schizophrenia. Despite the apparent lack of relation to joint morphogenesis and OA, common variation in *ASTN2* is robustly associated with both hip shape and OA [89, 115, 128]. Although a proposed mechanism relating to pain sensing and OA symptomatology has been suggested, this does not readily explain the shape association.

Although variation in genes identified in the studies outlined above, including *COL11A1*, *DOT1L*, *IHH*, *RUNX1*, *RETSAT*, and many others may also link joint development with subsequent OA, they have not been specifically included here, as they are yet to be established with both phenotypes at genome-wide significance.

## Conclusions

The study of increasingly large genome-wide meta-analyses together with multi-omics at scale, is starting to revolutionise our understanding of the heritable biology of osteoarthritis. We have established that 60% and 40% of susceptibility to hip and knee OA, respectively, is heritable. Current international initiatives, such as the GO Consortium https://www.genetics-osteoarthritis.com/home/index.html will further expand the depth and breadth of our understanding of the contribution of common and low-frequency variants to disease heritability. Study of the heritable biology of joint shape, and how it relates to OA risk, thus far, is yet to benefit from the same advantages of scale and phenotype specificity. Advances have been made in understanding the contribution of hip shape to OA risk, and shape variation can be measured with varying degrees of accuracy by several methods. However, for OA at other sites including the knee, clear shape phenotypes are less readily identified. The influence of other risk factors, such as sex, obesity and joint injury at the knee also make establishing relationships between joint shape and genotype more complex. Future abilities to link relevant imaging data to clinical and genomic datasets will narrow this gap.
